# Supplementary material for: The Development of 3D Primary Co-Culture Models of the Human Airway
Source: Int J Mol Sci. 2025 May 23;26(11):5027. doi: 10.3390/ijms26115027 (PMC12155322; doi:10.3390/ijms26115027)
Supplement: Supplementary file 1 [file ijms-26-05027-s001.zip › ijms-3564603-supplementary.pdf]

The development of 3D primary co-culture models of the human airway

Cinta Iriondo, Sem Koornneef, Kari-Pekka Skarp, Marjon Buscop-van Kempen, Anne Boerema-de Munck, Robbert J. Rottier

SUPPLEMENTARY FIGURES

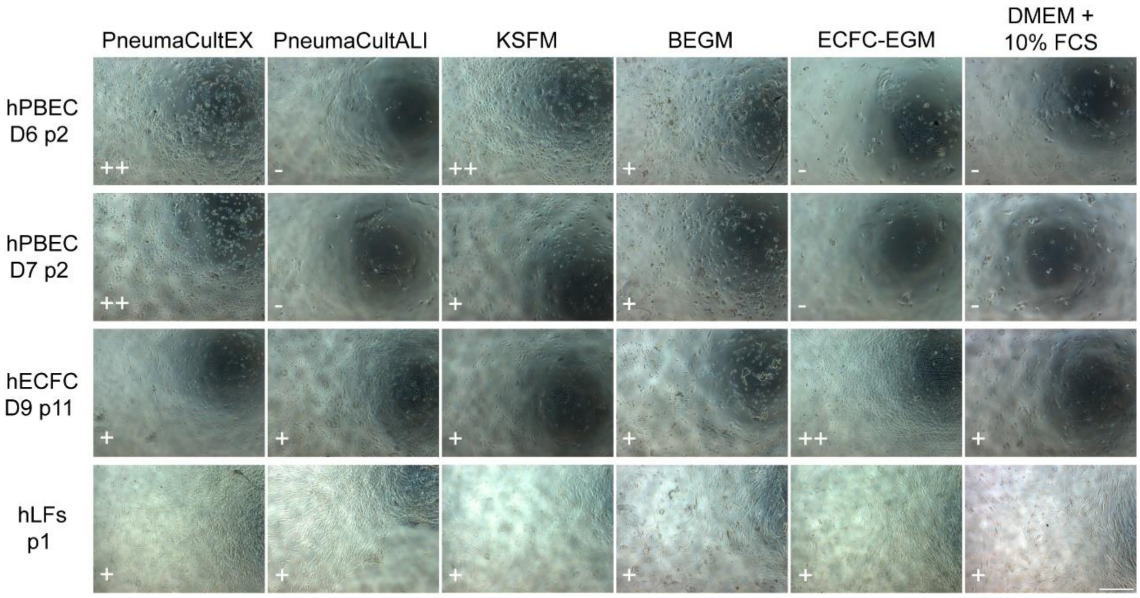

Figure S1. The effect of different media on cell morphology and proliferation.

Bright field images of different cultures with 2 independent hPBECs donors (D6, D7, p2), hECFCs (p11) and hLFs (p1) on day 3 of mono-culture in different hPBECs (PneumaCultEX, PneumaCultALI, KSFM, BEGM), endothelial (ECFC-EGM) and hLF (DMEM + 10 % FBS) media. HPBECs were plated at a $1*10^4$ cells/cm^2 density and hECFCs and hLFs at $5*10^3$ cells/cm^2 density. Scale bar = 200 μm.
Scoring is defined as: ++ very good (high confluency, minimal cell patches, proper cell morphology), + good (medium confluency, some cell patches, decent cell morphology), and - bad (low confluency, many cell patches, bad cell morphology).

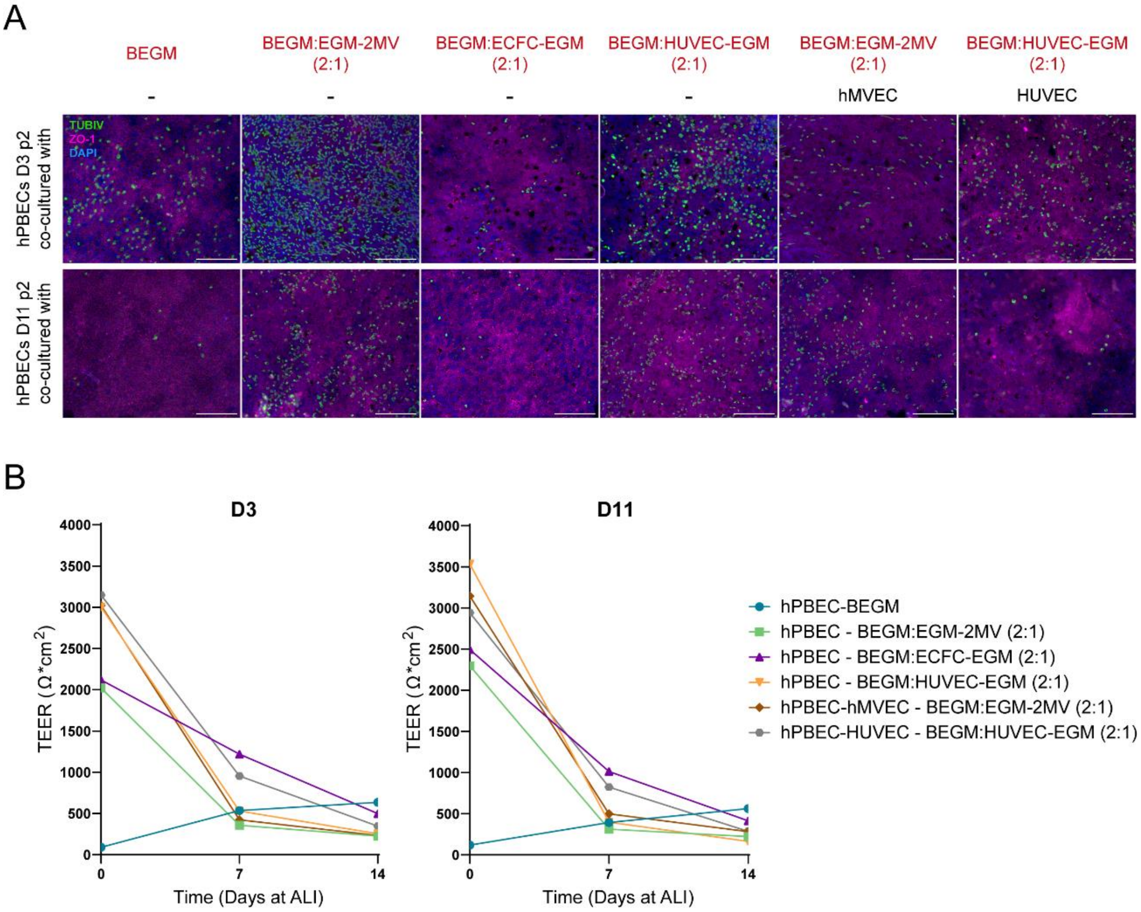

Figure S2. HPBECs differentiation is dependent on medium and cell types used.

A) Representative images of ALI cultures with hPBECs from two independent donors (Donor 3, p2 and Donor 11, p2) in different co-culture media (red), and as mono- or co-culture (hMVECs or HUVECs, black). HPEBCs were stained against cilia marker TUBIV (green), ZO-1 (magenta), and DAPI (blue) after 14 days at ALI. Scale bar = 200 µm. **B)** TEER values (Ω*cm^2) of ALI cultures shown in A and measured on days 0, 7 and 14 at ALI.

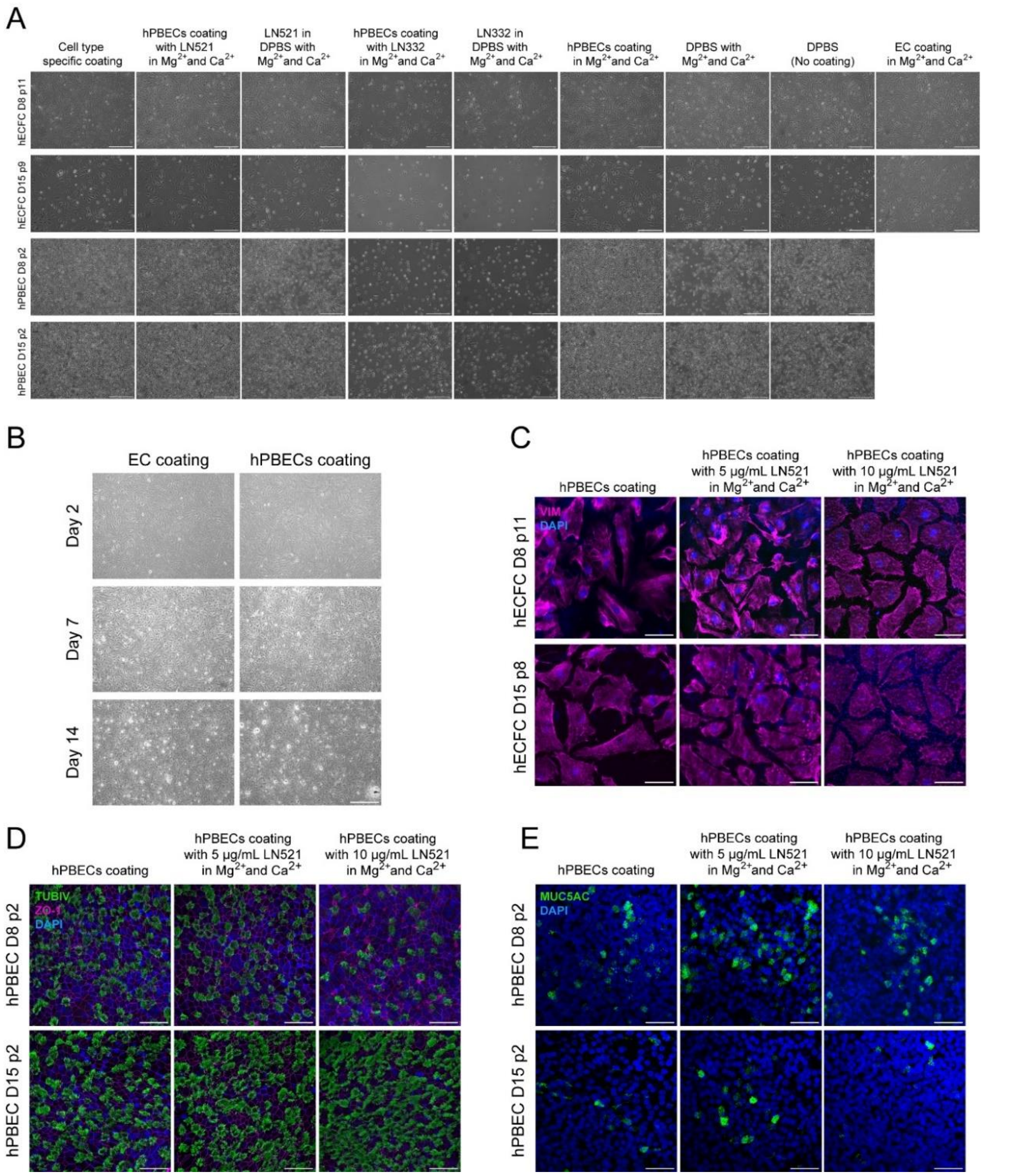

Figure S3. Testing Biolaminin 521 and 332 for hPBECs and endothelial cell coatings, and coatings for endothelial cells.

A) Bright field images of hPBECs and hECFCs (Donors 8 and 15) after 4 days of culture using different coating conditions involving biolaminin 521 (LN521) and 332 (LN332). Cells were plated at a density of $5*10^3$ cells/cm^2. Scale bar = 200 µm. **B)** Bright field images of hECFC (donor 7, p7) on inserts with either endothelial cell (EC) or hPBECs coating. ECFC-EGM medium was used and cells were plated at a density of $5*10^3$ cells/12 mm insert. Scale bar = 80 µm. **C-E)** Representative images of hPBECs-hECFC co-cultures (Donor 8 and 15) on inserts using different concentrations of LN521. Nuclei were stained using DAPI (blue). Scale bar = 50 µm. **C)** hECFCs (VIM, magenta), **D)** hPBECs cilia (TUBIV, green) and tight junctions (ZO-1, magenta), and **E)** hPBECs goblet cells (MUC5AC, green).

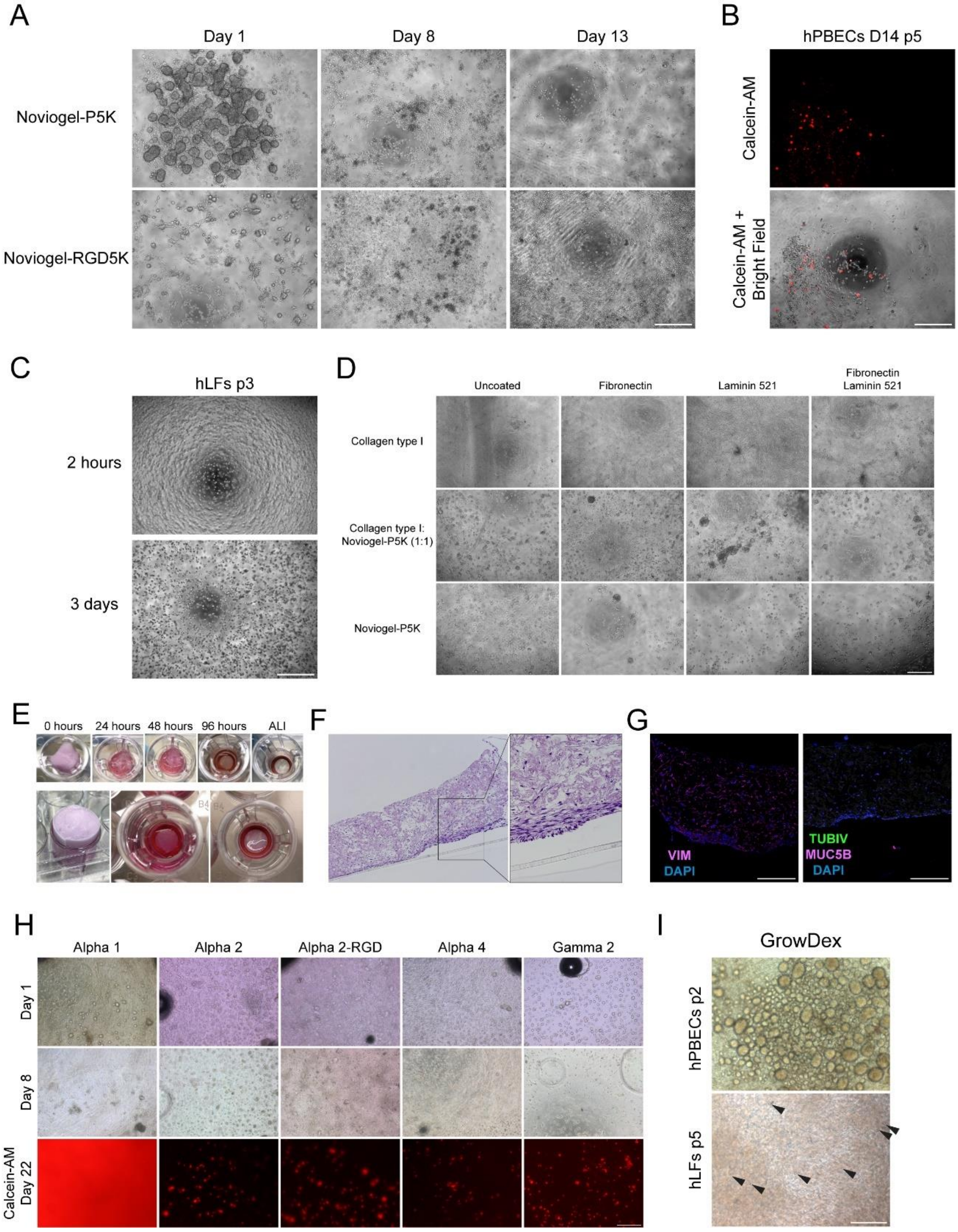

Figure S4. Synthetic gels used for the development of 3D human primary airway tri-culture. **A)** Bright field images of HPBECs (Donor 14, p5) plated on top of 2.18 mg/mL Noviogel-P5K or Noviogel-RGD5K. **B)** Calcein AM staining of viable hPBECs (Donor 14, p5) plated on top of 2.18 mg/mL Noviogel-RGD5K on day 17 of culture. **C)** Bright field images of hLFs p3 embedded in Noviogel-P5K. 10^5 cells /40 µL gel were used. **D)** Bright field images of hLFs p10 embedded in type I collagen gel (2.4 mg/mL PureCol, Advanced Matrix), Noviogel-P5K or a 1:1 mix of both gels. The gels were mixed with fibronectin and/or biolaminin 521. After 3 days, hPBECs (Donor 14, p3)

were seeded on top. **E-G)** HLFs were embedded in FN-4RepCT (FN-Silk; SpiderWeb foam) and then hPBECs (Donor 13, p3) were seeded on top. **E)** Images showing the generation process FN-Silk. **F)** H&E staining of a paraffin embedded membrane. **G)** IF images stained for mucociliary epithelium markers (TUBIV and MUC5B) and fibroblast marker (VIM). **H)** Bright field images of hPBECs plated on top of different types of Manchester Biogels. On day 22 fluorescent images were taken to look at viable cells with Calcein AM staining. **I)** Bright field images of hLF embedded in GrowDex hydrogel and hPBECs (Donor 14, p2) plated on top one day after culture. Black arrowheads indicate non-stretched hLFs. All scale bars = 200 μm.

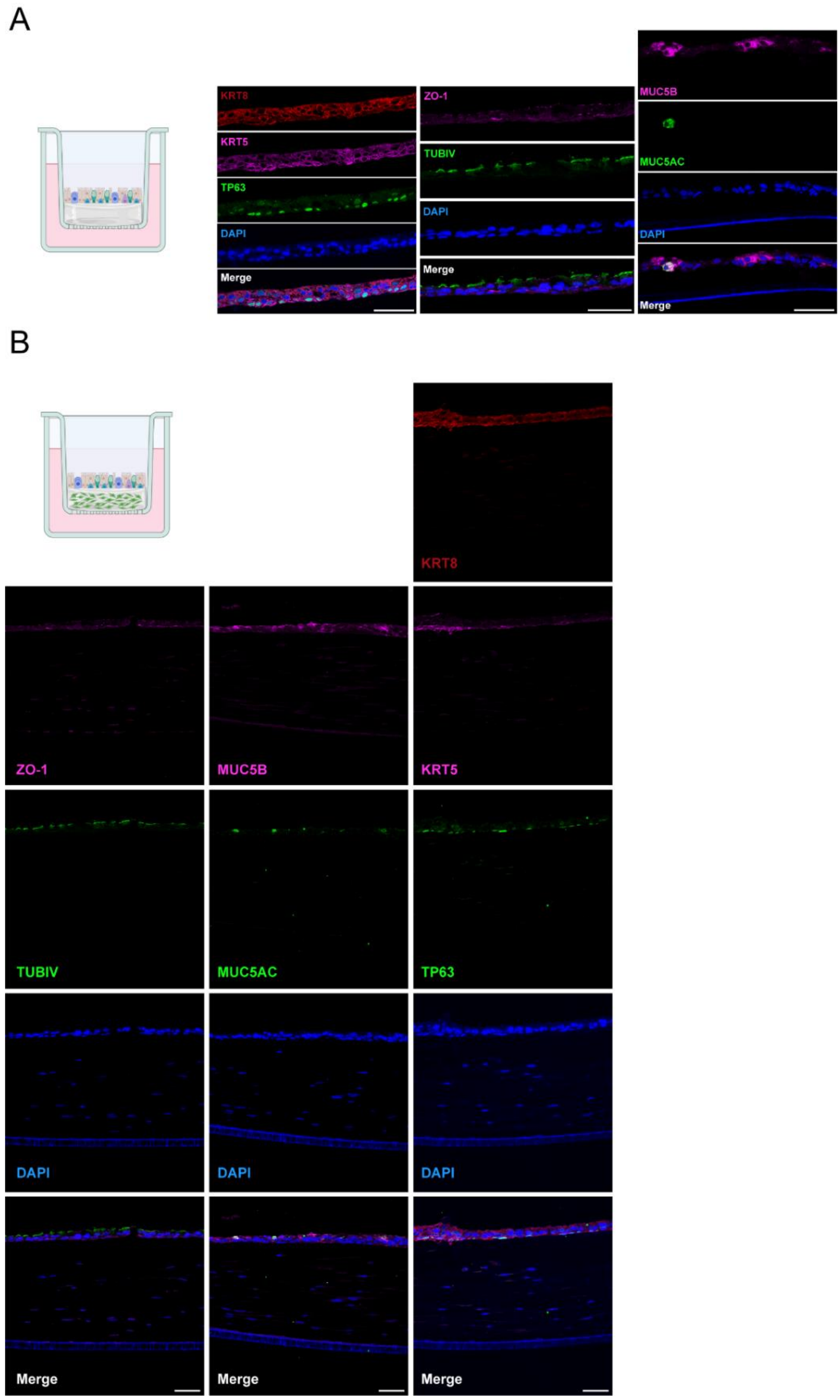

Figure S5. Additional images of hPBECs mono-cultures or hPBECs-hLFs on top of collagen gels.

A) Immunostaining of hPBECs mono-cultures on collagen, which also expressed basal (TP63, KRT5 and KRT8), ciliated (TUBIV), and goblet cells markers (MUC5AC and MUC5B), and tight junctions (ZO-1) after 14 days at ALI. Scale bar = 50 µm. **B)** Complementary images of Figure 4B showing nuclei of hLFs embedded in the collagen gel. Scale bar = 50 µm.

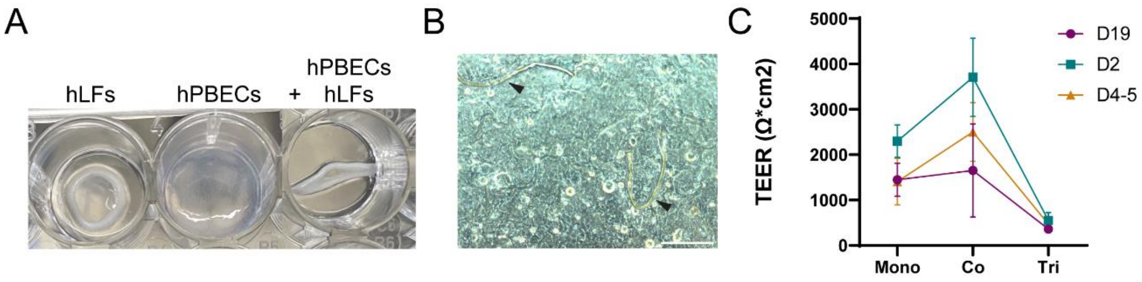

Figure S6. Drawbacks of using collagen gels for primary airway co-cultures.

A) Primary lung fibroblasts shrink the collagen gels. **B)** Remnants of the sterile gauze fibers. Black arrowheads indicate the fibers. Scale bar = 80 µm. **C)** TEER values after 14 days at ALI for mono-, co-, and tri-cultures (N=3, n=6).

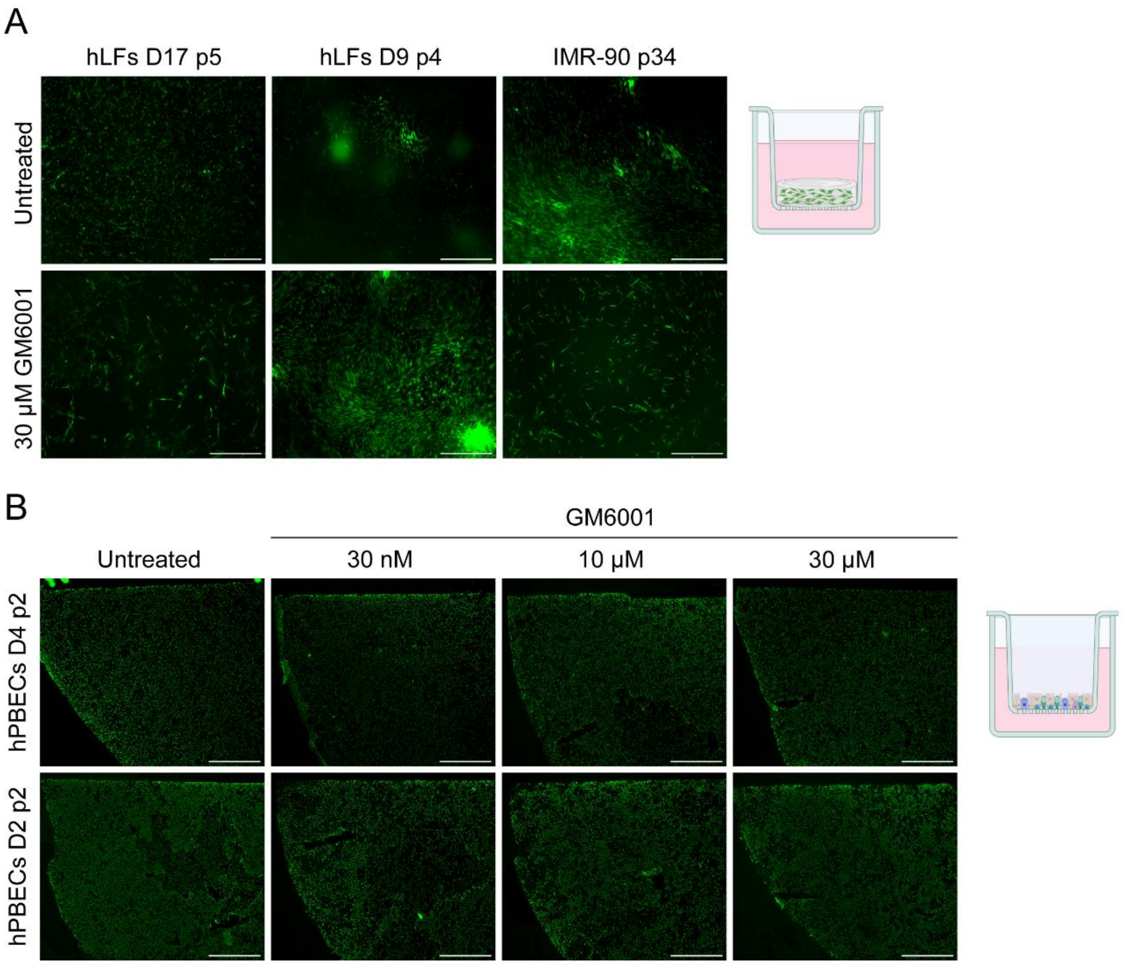

Figure S7. Testing the effects of broad-spectrum metalloprotease inhibitor GM6001 (Ilomastat) on hLFs for collagen gel contraction, and hPBECs differentiation.

A) Calcein AM images of viable hLFs and IMR-90 cell line (fetal lung fibroblasts) embedded in collagen gels with or without GM6001 on day 21 of culture. $1x10^4$ fibroblasts/gel were used. **B)** Images of ciliated (TUBIV, green) hPBECs after 14 days at ALI using different concentrations of GM6001. All scale bars = 1000 µm. Schemes were made using BioRender.com.

SUPPLEMENTARY METHODS

Table S1. Donors used in this study, their characteristics and lung cell types obtained.

Patient	hPBECs	hMVECs	hLFs	Sex	Age
Donor 1 (D1)	Yes	Yes	Yes	Female	66
Donor 2 (D2)	Yes	Yes	Yes	Female	52
Donor 3 (D3)	Yes	No	No	Female	63
Donor 4 (D4)	Yes	No	No	Male	73
Donor 5 (D5)	No	Yes	Yes	Male	72
Donor 6 (D6)	Yes	No	No	Male	45
Donor 7 (D7)	Yes	No	No	Male	61
Donor 8 (D8)	Yes	No	Yes	N/A	N/A
Donor 9 (D9)	Yes	No	Yes	N/A	N/A
Donor 10 (D10)	Yes	No	No	Male	51
Donor 11 (D11)	Yes	No	Yes	Female	56
Donor 12 (D12)	Yes	Yes	Yes	Male	71
Donor 13 (D13)	Yes	No	No	Male	N/A
Donor 14 (D14)	Yes	No	No	Male	61
Donor 15 (D15)	Yes	No	No	N/A	N/A
Donor 16 (D16)	No	Yes	No	Male	59
Donor 17 (D17)	Yes	No	Yes	Male	64
Donor 18 (D18)	No	No	Yes	Female	66
Donor 19 (D19)	Yes	Yes	Yes	Male	71

Table S2. All cell culture reagents and final concentrations used for hPBECs.

Name	Component	Final concentration	Supplier (Cat. No.)
hPBECs and co-culture coating	PureCol (PC) 3mg/mL	30 µg/mL	Advanced Biomatrix (5005)
	Human Fibronectin (FN)	10 µg/mL	EDM Millipore (FC010)
	Bovine Serum Albumin (BSA)	10 µg/mL	Sigma-Aldrich (A7030-10g)
	DPBS	1X	Sigma-Aldrich (D8537-500ML)
Complete KSFM medium (proliferation)	KSFM basal medium with L-glutamine	1X	ThermoFischer Scientific (17005)
	Bovine pituitary extract (BPE)	25 µg/mL	ThermoFischer Scientific (13028-014)
	Epithelial growth factor (EGF)	0.2 ng/mL	ThermoFischer Scientific (10450-013)
	Isoproterenol (IP)	1 µM	Sigma-Aldrich (I-6504-100 mg)
	P/S	1X (100 U/mL Penicillin and 100 µg/mL Streptomycin)	Sigma-Aldrich (P0781)
Complete BEGM medium (differentiation)	BEpiCM medium	1/2 X	ScienCell (3211)
	DMEM medium	1/2 X	StemCell (36250)
	2 x BEpiCGS supplement	1X	StemCell (3262)
	HEPES buffer	12.5 mM	Gibco, ThermoFischer Scientific (15630-056)
10X Soft Trypsin*	Difco Trypsin 1:250	0.3%	BD Biosciences (215240– 100g)
	EDTA	0.1%	Sigma-Aldrich (E1644-1KG)
	D-(+)- Glucose	1%	Sigma-Aldrich (G6152-100G)
	DPBS	1X	Sigma-Aldrich (D8537-500ML)
Soy Bean Trypsin Inhibitor (SBTI)	SBTI	1.1 mg/mL	Sigma-Aldrich (T9128-1G)
	KSFM basal medium with L-glutamine	1X	ThermoFischer Scientific (17005)
	P/S	1X (100 U/mL Penicillin and 100 µg/mL Streptomycin)	Sigma-Aldrich (P0781)

Table S3. Cell culture reagents and final concentrations.

Name	Component	Final concentration	Supplier (Cat. No.)
Endothelial cells (ECs)			
EC coating	Collagen R solution (0.4%)	50 µg/mL	Serva (47256.01)
	Glacial Acetic acid in sterile water	0.02 N	Fischer Scientific (17.4 N, 505216)
Trypsin-EDTA (TE) solution	TE solution*	1X	Sigma-Aldrich (T3924)
hMVEC and hMVEC-L (passage 3-8)			
Complete EGM-2MV medium	EGM-2MV medium	1X all supplements	Lonza (CC-3202)
	P/S	1X (100 U/mL Penicillin and 100 µg/mL Streptomycin)	Sigma-Aldrich (P0781)
hECFCs (passage 3-12)			
Complete ECFC-EGM medium	EGM medium	1X all supplements but EGF	Lonza (CC-3162)
	10% FBS	N/A	Capricorn Scientific (FBS-12A)
	P/S	1X (100 U/mL Penicillin and 100 µg/mL Streptomycin)	Sigma-Aldrich (P0781)
HUVECs (passage 3-8)			
HUVEC-EGM	Same as ECFC-EGM, but containing the EGF supplement		
hLFs (passage 1-8)			
Complete DMEM medium	DMEM, (1X), high glucose, pyruvate	N/A	Gibco, Thermofisher Scientific (41966-029) Capricorn Scientific (FBS-12A)
	10% FBS	N/A	Capricorn Scientific (FBS-12A)
	P/S	1X (100 U/mL Penicillin and 100 µg/mL Streptomycin)	Sigma-Aldrich (P0781)
Trypsin-EDTA (TE) solution	TE solution*	1X	Sigma-Aldrich (T3924)

***Note:** Endothelial cells and hLFs TE solutions were quenched with their own media or 10% FBS in DPBS.

Table S4. H&E staining protocol for tri-cultures paraffine sections.

Step	Duration	Comments	Supplier (Cat. No)
Xylene	3 min	Use different containers for each step to dilute the components. Soak all the slide in the containers using a slide holder. Make sure that the container and slide holder plastic do not get dissolved in xylene. EtOH is diluted in MilliQ water.	VWR (vwrk4055-9005)
Xylene	3 min		
Xylene	3 min		
100% EtOH	2 min		Ethanol absolute: Honeywell (32221) or VWR (83813.360)
100% EtOH	2 min		
96% EtOH	2 min		
70% EtOH	2 min		
50% EtOH	2 min		
Tap water	2 min	Not running water	N/A
Hematoxylin	4 min	Protect from light	VWR (VWRK4085.9001)
Tap water	7 min	Running: fill the container with water and apply a gentle stream of water. Let it overflow in a sink.	N/A
Eosin	3 min	Protect from light	VWR (10047101)
Tap water	45 s	Running water	N/A
70% EtOH	10 s	Use different containers for each step to dilute the components. Soak all the slide in the containers using a slide holder. Make sure that the container and slide holder plastic do not get dissolved in xylene. EtOH is diluted in MilliQ water.	Ethanol absolute: Honeywell (32221) or VWR (83813.360)
80% EtOH	10 s		
96% EtOH	10 s		
100% EtOH	1 min		
100% EtOH	2 min		
Xylene	3 min		VWR (vwrk4055-9005)
Xylene	3 min		
Pertex	N/A	Mount slides using pertex and then place a coverslip on top. Let samples dry overnight at RT and store samples at RT.	Klinipath (am-0811)

Note: H&E protocol should be performed inside a fume hood as xylene fumes are toxic and smelly.

Table S5. Primary and secondary antibodies used and their characteristics.

Primary Antibody	Dilution insert membranes and Whole Mount tri-cultures	Dilution paraffin sections	Supplier	Cat. No.
TUBIV, mouse monoclonal	1:200	1:100	BioGenex	MU178-UC
FOXJ1, goat polyclonal	1:100	Does not work	R&D systems	AF3619
ZO-1, rabbit polyclonal	1:100	1:50	Zymed, Invitrogen	40-2300
MUC5AC, mouse monoclonal	1:500	1:500	Abcam	ab3649
MUC5B, rabbit polyclonal	1:500	1:500	Sigma-Aldrich	HPA008246
TP63, mouse monoclonal	1:100	1:50	Abcam	ab735
KRT5, rabbit polyclonal	1:500	1:500	ITK diagnostics BV, BioLegend	905501
KRT8, rat monoclonal	1:100	1:100	DSHB	TROMA-I
CD31 (PECAM-1), mouse monoclonal	1:100	Does not work	BioLegend	303101
ERG-1, rabbit monoclonal	1:500	1:500	Abcam	ab92513
VIM, rabbit monoclonal	1:500	1:500	Abcam	ab92547
aSMA, mouse monoclonal	1:1000	1:800	Fisher Scientific (Epredia)	MS113P1
**Secondary Antibody**				
Alexa Fluor ®488 Donkey anti Mouse IgG	1:500	1:500	Jackson ImmunoResearch	715-545-151
Alexa Fluor ®594 Donkey anti Rabbit IgG	1:500	1:500	Jackson ImmunoResearch	711-585-152
Alexa Fluor ®594 Donkey anti Rat IgG	1:500	1:500	Jackson ImmunoResearch	712-585-153
Alexa Fluor ®594 Donkey anti Goat IgG	1:500	1:500	Jackson ImmunoResearch	705-585-147
Alexa Fluor ®647 Donkey anti Rabbit IgG	1:500	1:500	Jackson ImmunoResearch	711-605-152

Table S6. Whole mount immunofluorescence staining of tri-cultures protocol.

Step	Whole mount		Insert membranes	
	Time	Manner	Time	Manner
Fixation 4%PFA	1 hour	RT	10 min	RT
Wash 0.1% PBS-T	10 min	3 times	-	3 times
Blocking Buffer	1 hour	RT	30 min	RT
Primary Ab	ON	4 °C, 24-well, 400 μL	ON	4 °C, humidity chamber
Rinse 0.03% PBS-T	-	3 times	-	3 times
Wash 0.03% PBS-T	30 min	3 times	10 min	3 times
Secondary Ab + DAPI	3 hours	RT, dark, 24-well, 400 μL	2 hours	RT, dark, 48-well, 200 μL
Rinse 0.03% PBS-T	-	3 times	-	3 times
Wash 0.03% PBS-T	30 min	3 times	10 min	3 times
DPBS	10 min	1 time	-	1 time

Immunofluorescence staining of insert membranes also added as a reference.
